# Cellulases from Thermophiles Found by Metagenomics

**DOI:** 10.3390/microorganisms6030066

**Published:** 2018-07-10

**Authors:** Juan-José Escuder-Rodríguez, María-Eugenia DeCastro, María-Esperanza Cerdán, Esther Rodríguez-Belmonte, Manuel Becerra, María-Isabel González-Siso

**Affiliations:** Grupo EXPRELA, Centro de Investigacións Científicas Avanzadas (CICA), Departamento de Bioloxía, Facultade de Ciencias, Universidade da Coruña, 15071 A Corunna, Spain; j.escuder@udc.es (J.-J.E.-R.); m.decastro@udc.es (M.-E.D.); esper.cerdan@udc.es (M.-E.C.); esther.belmonte@udc.es (E.R.-B.); manuel.becerra@udc.es (M.B.)

**Keywords:** cellulases, thermophiles, metagenomics, biotechnology

## Abstract

Cellulases are a heterogeneous group of enzymes that synergistically catalyze the hydrolysis of cellulose, the major component of plant biomass. Such reaction has biotechnological applications in a broad spectrum of industries, where they can provide a more sustainable model of production. As a prerequisite for their implementation, these enzymes need to be able to operate in the conditions the industrial process requires. Thus, cellulases retrieved from extremophiles, and more specifically those of thermophiles, are likely to be more appropriate for industrial needs in which high temperatures are involved. Metagenomics, the study of genes and gene products from the whole community genomic DNA present in an environmental sample, is a powerful tool for bioprospecting in search of novel enzymes. In this review, we describe the cellulolytic systems, we summarize their biotechnological applications, and we discuss the strategies adopted in the field of metagenomics for the discovery of new cellulases, focusing on those of thermophilic microorganisms.

## 1. Introduction

Cellulose is a complex polymer that can be hydrolyzed into glucose by the synergetic action of a mixture of enzymes known as cellulases. Plants fix atmospheric CO_2_ and incorporate about half of the carbon in structural polysaccharides and lignin (lignocellulose). This structural carbon can be used as an energy source by cellulolytic microorganisms [1]. The cellulolytic enzymes can form an enzyme complex known as the cellulosome, in which they are anchored to a common scaffold. This structure is mostly observed in anaerobes and exclusively in bacteria. They can also act as non-complexed extracellular free cellulase systems, more often associated to aerobes and present in fungi, bacteria, and archaea [1,2,3,4]. Additionally, other auxiliary enzymes like lytic polysaccharide monooxygenases have been reported to also contribute to the degradation of cellulose by cellulases by enhancing their activity [5,6,7]. An enhancer effect has also been proposed for hemicellulases such as xylanases, mannanases, galactosidases, and β-1,3-1,4-glycanases, which has activity on polysaccharides present in plant biomass by allowing cellulases to better reach the substrate [8].

Microorganisms adapted to live in harsh conditions (from a human standpoint) are known as extremophiles. Their enzymes, and especially the extracellular ones, have adopted mechanisms to maintain their function in such environments and are known as extremozymes. They are interesting from a biotechnological perspective, as many industrial applications involve conditions similar to those of extreme environments, and a more sustainable production model would require biocatalysts able to operate in such conditions [9,10,11].

Thermophiles are extremophiles that thrive at high temperatures ranging from moderate thermophiles (capable of growth at temperatures between 50 °C and 64 °C), extreme thermophiles (between 65 °C and 79 °C), and hyperthermophiles (over 80 °C) [12]. Extreme habitats where these microorganisms can be found include deep-sea hydrothermal vents, hot springs, volcanic fields, mud pots and deserts, and human-made environments like compost, among others. Many enzymes of industrial importance have been retrieved from thermophiles, including cellulases [11].

## 2. Modular Structure of Cellulases and their Classification

Most cellulases have a modular design, in which two or more discrete units have cooperative functions and are connected through linker sequences. Usually, this modular design includes the catalytic domain linked to a carbohydrate-binding module (CBM), but other non-catalytic domains can also be present, and multiple catalytic domains or CBMs can exist on the same enzyme. The CBM helps in the catalytic process by increasing the concentration of the enzyme near the polysaccharides they bind [5,13,14] and by disrupting the crystalline cellulose structure, increasing substrate accessibility [15]. As previously stated, some cellulases can form the enzyme complex known as the cellulosome, where they are anchored to a protein scaffold (composed of non-catalytic proteins known as scaffoldins). These cellulases contain dockerin domains that bind to the cohesin module of the scaffoldins, although these domains have also been described in proteins not related to the cellulosome [16]. In cellulosomes, the scaffolding proteins might also contain CBM modules [17].

**Figure 1:**
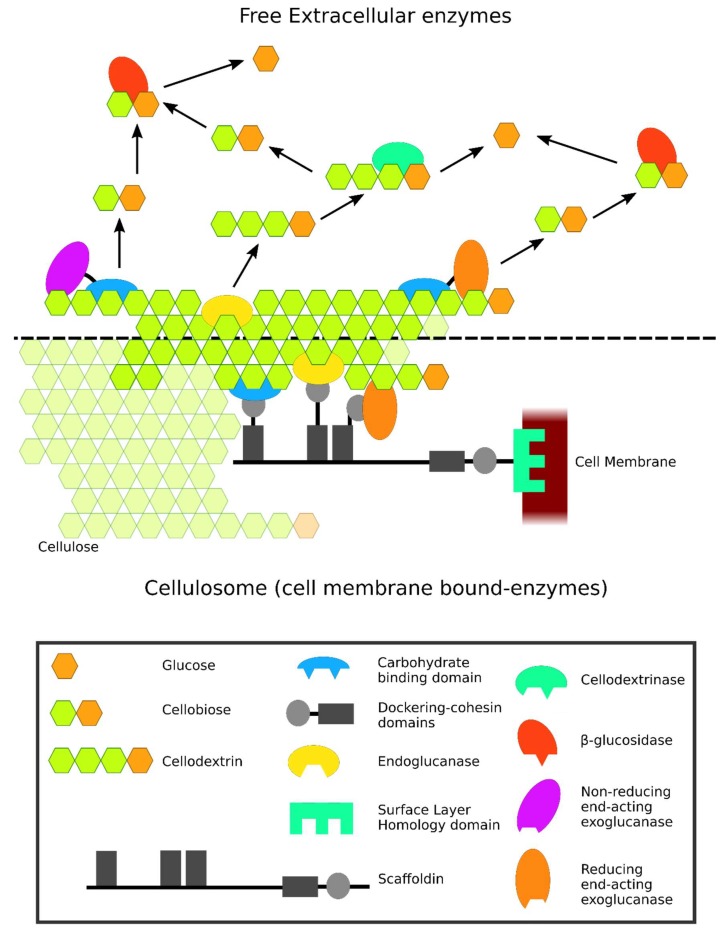
Overview of the two strategies (free or cell-bound cellulase systems) for degrading cellulose. In free extracellular systems, endoglucanases and exoglucanases act synergistically, with the endoglucanase cutting amorphous cellulose providing chain ends for exoglucanases to release cellobiose. Then, β-glucosidases complete the process of cellulose hydrolysis by releasing glucose. Also, cellodextrins released by endoglucanases can be further hydrolysed by cellodextrinases. The carbohydrate binding domain directs the enzymes to their specific substrates. In the cellulosome system, all cellulases are anchored to a common scaffold but are generally thought to follow the same synergic mode of action. The scaffolding is bound to the cell membrane through the surface layer homology domain, while a network of dockerin and cohesin domains amplifies the number of cellulases bound to the same scaffolding unit. Lastly, a carbohydrate binding domain is responsible for the targeting of the whole complex to the substrate.

The classic classification of cellulases is based on the mechanism of action of their catalytic domains and on their substrate specificity. This classification allows us to distinguish three major types of cellulases: β-1,4-endoglucanases (EC 3.2.1.4), exoglucanases [non-reducing end cellobiohydrolases (EC 3.2.1.91), reducing-end cellobiohydrolases (EC 3.2.1.176) and cellodextrinases (EC 3.2.1.74)], and β-glucosidases (EC 3.2.1.21) [3,7,18]. Endoglucanases act randomly cleaving internal glycosidic bonds of cellulose chains, releasing oligosaccharides of different length (like cellobiose and cellotriose). Cellobiohydrolases act processively on the reducing and non-reducing ends of cellulose, primarily releasing cellobiose but also other short oligosaccharides. Cellodextrinases act on soluble cellooligosaccharides, also releasing cellobiose. Lastly, β-glucosidases perform the hydrolysis of cellodextrins and cellobiose into glucose, enhancing both endoglucanase and exoglucanase activities by reducing the end product inhibition [3,6,7,9]. A schematic representation of cellulases acting on cellulose is depicted in Figure 1.

Due to the enormous variety of polysaccharides that exist in nature, and the fact that cellulases are not always easy to categorize as only endo- or exo-acting enzymes [19], an alternative classification based on amino acid sequence similarity was proposed [20]. Rather than substrate specificity, this classification addresses the structure-function relationships, substrate recognition and enzymatic reaction mechanisms, and evolutionary relationships between the enzymes. The publicly available Carbohydrate-Active Enzymes Database (CAZy, http://cazy.org) contains the classification of glycoside hydrolase (GH) families in which the cellulases are included. The database at the time of writing lists 149 different GH families [21]. Endoglucanases are mainly present in 12 GH families: GH5-9, GH12, GH44, GH45, GH48, GH51, GH74, and GH124; cellobiohydrolases acting on non-reducing ends can be found in families GH5, GH6, and GH9, whereas the reducing-end acting ones are mostly present in GH7, GH9, and GH48; cellodextrinases are distributed in families GH1, GH3, GH5, and GH9; and, lastly, β-glucosidases belong in families GH1-3, GH5, GH9, GH30, GH39, and GH116 [20].

Even if they share structural characteristics, members of the same GH family may differ widely in substrate specificity and their evolutionary history, and, due to their multidomain nature, some enzymes may contain sequences from different GH families [3,6,10]. As a further classification for GHs, some families are also grouped in clans in regard to their folding, as it is more conserved than their amino acid sequence [14]. Clans are designated by a letter, and some cellulases fall inside these groups: GH-A (with a (β/α)_8_ barrel) includes cellulases from families GH1, GH2, GH5, GH30, GH39, and GH51; GH-B (that fold in β-jelly roll) contains family GH7; GH-C (also folding with a β-jelly roll) includes family GH12; GH-M (folding with a (α/α)_6_ barrel) comprises families GH8 and GH48; and GH-O [(α/α)_6_ barrel folding] contains family GH116.

In regard to the catalytic mechanism, GHs (including cellulases) may perform the hydrolysis of the glycosidic bond by an inverting or retaining mechanism, whether the configuration of the substrate’s anomeric carbon (C1) is changed or not after the cleavage. Retaining enzymes have a double nucleophilic displacement mechanism involving two carboxylate catalytic residues. Inverting enzymes act with a single nucleophilic displacement mechanism, also involving two carboxylate catalytic residues [1]. For cellulases, seven GH families have an inverting mechanism of catalysis (6, 8, 9, 45, 48, 74, and 124), whereas eleven act with a retaining mechanism (1–3, 5, 7, 12, 30, 39, 44, 51, and 116) [20,22].

## 3. Factors Influencing Thermostability of Thermophile Cellulases

As pointed out, a greater half-life of cellulases at high temperatures is a desirable trait for many industrial applications. In order to obtain more thermostable variants of cellulases, the molecular mechanisms behind thermostability have been studied. Some researchers argue that the study of smaller, single-domain enzymes would make it easier to pinpoint the mechanisms involved in a higher resistance to high temperature [23], while others have studied the effect of the number of domains and linker sequences and domain-removal on thermostability, though opposing stabilizing and destabilizing effects have been described in this regard [5].

Several stabilization factors have been proposed for the increased thermostability of thermozymes, such an increased number of ion pairs, a lower number of loops and cavities (thus making the protein more compact), a reduced ratio of protein surface area to protein volume, a higher number of proline residues in loops (limiting the conformational freedom of the protein), an increased amount of hydrophobic interactions, and a greater degree of oligomerization [24,25]. Despite that, a direct correlation between all these factors and protein thermostability cannot always be established; for example, for *Humicola insolens* exoglucanase Cel6A the addition of proline residues in the loop regions did not achieve greater stability and in some instances had the opposite effect [26]. It has been also proposed that proteins can undergo structure-based or sequence-based stabilization strategies through evolution. As thermophilic archaea emerged in already extreme environments, their enzymes would initially favour stable folding at high temperatures, whereas thermophilic bacteria would have to enhance the thermostability of their proteins by point mutations that increase the number of ion-pairs in order to colonize the new habitats. Despite this theory, it has been found that among archaea, the two different stabilization models can be adopted [24].

There are also reports on how hydrophobic and aromatic residues can play a major role in protein thermal stability, like in the endoglucanase from family GH12 from *Aspergillus niger* [27]. Other authors have described an increased percentage of the charged amino acid glutamic acid in thermophilic enzymes from family GH12 compared to mesophilic ones, which is thought to stabilize the protein’s structure through salt bridges and hydrogen bonds [23]. Moreover, some key residues for protein stability have been already identified in this protein family [1]. When comparing mesophilic and thermophilic exoglucanases from family GH7, the potential disulphide bridge formation by the presence of cysteine residues could not be linked to an increased thermostability, whereas a higher number of charged residues and lower number of polar residues was observed in the more thermostable enzymes [28]. However, it was found that rational mutagenesis introducing disulphide bridges in an exoglucanase from this family did allow the mutant proteins to be more thermostable [29].

Lastly, eukaryotes’ post-translational modifications (including glycosylation, phosphorylation, acetylation, and methylation) have been reported to account for protein thermostability [27], and heterologous expression of the enzyme in a yeast host can be a desirable production system for industrial applications.

The yeast *Pichia pastoris,* in particular, has been extensively employed due to this property, along with its relative ease for genetic manipulation and high level of protein expression [19,30,31,32], coupled with inexpensive production media and relatively simple protein processing protocols [33]. Nevertheless, most studies regarding the discovery and the characterization of new thermophilic cellulases have involved the model organism *Escherichia coli* [34,35,36,37,38], sometimes at the expense of thermostability [39].

## 4. Biotechnological Applications by Thermophile Cellulases

Thermozymes have general advantages over their mesophilic counterparts in regard to their application in various industries, as they are generally more stable towards extreme temperatures and pH, as well as in the presence of chemically destabilizing agents, and function at high temperatures with higher reaction rates [35] and higher mass-transfer rates that increase the substrates’ solubility, as well as a lower risk of contamination [27]. Lastly, the process design gains flexibility (e.g., current process configurations with operations that needed pre-treatment of the substrates to lower the temperature can now be performed simultaneously without the requirement of a temperature modification between them), which in turn can reduce the cost of operation [27]. On the other hand, and as previously stated, preferred systems to produce these enzymes are not thermophilic, as thermophile production faces many technical challenges due to limited knowledge of their physiology and genetics, difficulty of growing and not being Generally Recognized As Safe [27] as defined by the US Food and Drug Administration under sections 201(s) and 409 of the Federal Food, Drug, and Cosmetic Act. In regard to the production process, extracellular enzymes are desirable, as they are easier to purify [27,33].

The range of industries in which degradation of cellulose by cellulases is required is considerably wide and includes biofuels (conversion of plant biomass in bioethanol), food and brewing, textiles (biostoning and biopolishing), laundry (in detergent formulations), pulp and paper (biopulping), and animal feeds [35]. Other uses include waste management, improvement of soils for agriculture [40], and extraction of compounds from plants such as olive oil, pigments, and bioactive molecules [4].

The full conversion of cellulose into glucose, which can later be converted into ethanol (named bioethanol to stress it being a biofuel, in contrast with the classic fossil fuels) has been previously stated to require the combined action of multiple cellulolytic enzymes (endo- and exoglucanases and β-glucosidases). This process has gained a lot of interest, as plant biomass poses a promising renewable substrate alternative to assess the increasing energy demands while limiting the use of fossil fuels [2,41]. In this regard, the use of non-food lignocellulosic waste from agriculture and forestry has replaced food crops as the substrate of choice, as the use of the latter would have the associated risk of raising basic foods prices and limiting their supply [42]. In general, biorefining (using biomass as a substrate to produce fuels, energy, or chemicals) benefits from thermostable enzymes, as heat treatment, is an important step for the pre-processing of the lignocellulosic material [43,44,45]. The use of thermostable cellulases for the treatment and pretreatment of the biomass reduces the energy cost of the process, improves the solubility of the substrate, reduces its viscosity, and reduces dependency on the use of environmentally harsh chemicals [39,45].

### 4.1. Endoglucanase-Specific Industrial Applications

Endoglucanases have been used in the textile industry for the process called biostoning. Biostoning achieves a wash-down look on denim cotton clothes, and represents an alternative to the chemical method using pumice stone. Biostoning has a number of advantages over the classical method, such as greater yields, less labor-intensive operations, more secure workplace, shorter time requirements, lower damage to the machinery, and a more environmentally friendly process [4].

Another textile industrial process in which endoglucanases are employed is the biopolishing of cotton products. This process removes the microfibrils from cottons’ surfaces, enhancing the colour brightness and making them more resistant to pilling [40], as well as softening the product [46] and giving it a cleaner and smoother look [4]. Biopolishing is often performed after another enzymatic process called desizing (in which amylases remove starch from the fabrics). Desizing uses temperatures higher than 70 °C, so endoglucanases operating at such temperatures would be interesting for combining both processes and thus reducing the required time and energy costs [46]. Other textile processes in which endoglucanases are employed to remove cellulosic impurities, replacing chemical treatments, include bio-carbonization of polyester-cotton blends, wool scouring, and de-fibrillation of Lyocell [4].

In the brewing industry, the production of malt generates high molecular weight β-glucans. The presence of these molecules increases viscosity, lowering the efficiency and yield of the process due to the increased difficulty for pumping and also making filtration difficult [33]. As such, the addition of endoglucanases would alleviate those problems, allowing for the hydrolysis of β-glucans [33]. Also, endoglucanases may be used to increase the extraction of fermentable compounds both in brewing and fermentation industries [47].

In the laundry industry, the use of endoglucanases in detergent formulations is known to improve the colour brightness and soften cotton fabrics [4], similarly to the biopolishing in the textile industry.

In the animal feed industry, they enhance β-glucan digestibility and nutrient bioavailability [47], and have been shown to increase weight gain and milk production of ruminants [4].

Endoglucanases have been extensively used in the pulp and paper industry for the treatment of pulp wastes [4,47], deinking and removal of pollutants from paper without altering its brightness and strength [4], and in the pulping process (bio-pulping), reducing the energy cost of the process and improving the beatability of the pulp [4]. 

### 4.2. Exoglucanase-Specific Industrial Applications

As in nature, efficient degradation of cellulose from biomass in industrial applications requires the synergic action of a mixture of cellulases [26,48]. Synergism has been described between endoglucanases and exoglucanases, between reducing-end-acting and non-reducing-end-acting exoglucanases, between processive endoglucanases and endo- or exoglucanases, and between β-glucosidases and the other cellulases [48]. As such, the previously described industrial applications benefit from the addition of exoglucanases to enzyme mixtures already containing other cellulase classes.

### 4.3. β-glucosidase-Specific Industrial Applications

In addition to their application in the last step of cellulose hydrolysis to release glucose, β-glucosidases have several additional biotechnological applications.

In the food industry, they can be used to release aromatic compounds from fruit and fermentation products [49], like the release of terpenoids and phenylpropanoids in wine to enhance its aroma [50,51]. Other uses include juice clarification [32] and hydrolysis of bitter compounds in its extraction [52], and, in general, improvement of quality of beverages and foods [44] including colour, aroma, flavour, texture, and nutritional value [4].

In the pharmaceutical industry, they are used to deglycosylate ginsenosides, active compounds with many pharmaceutical uses, as the natural glycosylated ginsenosides from ginseng root are less active and less absorbable [50,52,53]. Similarly, they are used to convert the bioactive isoflavonoid-glucosides from soybean and other leguminous plants into aglycones with higher bioavailability and pharmaceutical activity [44,50,54]. Moreover, β-glucosidases can perform reverse hydrolysis or transglycosylation catalytic pathways for the formation of new glycosidic bonds, a property that makes them interesting for the production of functional compounds, and nutraceutical and pharmaceutical products [44]. For example gentibiose, a product of transglycosylation by β-glucanases, can be used as a prebiotic food additive [50]. These kinds of enzymatic transformations constitute important alternatives to chemical synthesis involving the use of organic solvents [55]. In this regard, the valorization of spent coffee grounds to produce isoflavone glycosides has also been proposed [54].

## 5. Metagenomics for the Search of Novel Cellulases

The metabolism of thermophiles holds great potential for several industrial applications, but due to the difficulty of growing extremophiles in the laboratory, culture-independent techniques constitute instrumental methods to have access to it. The use of metagenomics, the study of whole communities’ genomes, has proven to be a useful tool for the discovery of novel cellulases, both in the functional and the sequence-based approaches [10,11]. Several studies had found cellulases in a wide variety of natural thermophilic environments, such as hydrothermal vents [56,57], continental geothermal pools and hotsprings [58,59], and man-made environments like vermicompost [60], compost [37,61,62], and biogas digesters [63]. Nevertheless, high-temperature acting enzymes have also been found by metagenomics on moderate-temperature samples like soils [40,64,65] and aquatic environments [66], and in microorganisms associated with animals like microbial communities in rabbit cecum [67], ruminants rumen [36,68,69], earthworm casts [70], and thermite guts [71,72].

The main limiting factor for the discovery of new thermophile cellulases by functional metagenomics is the host organism used for the metagenomic libraries, typically the mesophilic bacterium *E. coli*, which may have a limited or biased expression of gene products from thermophiles [3]. One of the proposed solutions for this problem is the use of an alternative thermophilic host for the metagenomic libraries that would increase the hit detection rate for cellulases [11]. It should also be noted that bacteria hosts are not able to express fungal enzymes, as the promoter and intron regions are not recognized [3]. Lastly, the discovery of novel cellobiohydrolases through metagenomics is limited due to the lack of specific substrates other than AVICEL that can discriminate between true cellobiohydrolases and other celullases, as AVICEL has the requirement of a synergy between an endoglucanase and an exoglucanase for detection of activity [3]. The other metagenomic approach, an analysis of the whole metagenome sequencing data, can overcome the problems that arise in the expression-based approach. Regardless, the discovery of gene products with novel characteristics is hindered due to the need of high amino acid homology with already known enzymes, and before assigning putative proteins a function, activities should be verified [11].

## 6. Thermophile Cellulases Characterized

Table 1, Table 2, Table 3, Table 4 and Table 5 list, respectively, endoglucanases, exoglucanases acting on non-reducing ends, exoglucanases acting on reducing ends, cellodextrinases and β-glucanases that can be considered thermophilic (optimum temperature at 50 °C or higher), and other key parameters for their industrial application, namely, pH optimum and temperature stability, their classification according to the CAZY database, and their source organism.

## 7. Conclusions

Cellulases retrieved from high-temperature environments are considered a valuable industrial resource for their vast biotechnological potential [35]. The use of culture-independent techniques such as metagenomics has allowed us to discover enzymes from unknown microorganisms thriving in extreme habitats [11]. Since the last decade, metagenomics has led to the discovery of almost half (46%) of the characterized thermophilic endoglucanases (Table 1) described in that period and a fraction (17% of each total) of the thermophilic cellobiosidases acting on the non-reducing end of cellulose (Table 2) and thermophilic β-glucosidases (Table 5). Nevertheless, metagenomics have yet to yield thermophilic cellobiosidases acting on the reducing end of cellulose (Table 3) or thermophilic cellodextrinases (Table 4). The lack of enzymes found by this strategy is likely a consequence of the mechanism of action of those enzymes, as the lack of substrates specific to those activities greatly limits its positive hit ratio. While thermophilic β-glucosidases discovered in the last 5 years still account for a similar proportion of the total (15%), no more thermophilic cellobiosidases acting on non-reducing ends have been characterized by this method. On the other hand, the proportion of thermophilic endoglucanases that have been characterized and identified by metagenomics have grown to account for more than half of the total (55%) in the last 5 years. In total, almost one fifth (18%) of all the thermophilic cellulases identified and characterized so far have been found by metagenomics. Functional metagenomic bottlenecks, like the lack of substrates for specific cellulases and problems associated with heterologous expression [3], and validation of sequence-based metagenomics annotation of cellulases [11], still need to be addressed to further increase the number of cellulases identified using these strategies. Biomining for novel thermophilic cellulases through metagenomic means is thus an ongoing challenge, with great potential as a source of commercially and environmentally important byocatalysts in all sorts of biotechnological applications.

## Figures and Tables

**Table 1:** Characterized endoglucanases (EC 3.2.1.4) from thermophiles. NM: not measured.

Enzyme	GH Family Domains	Optimum Temperature	Optimum pH	Temperature Stability ^1^	Source	Reference
EGPh	5	>97 °C	5.4–6.0	80%; 97 °C; 3 h	Archaea (*Pyrococcus horikoshii*)	[46]
EG1	5	83 °C	5.0	20%; 90 °C; 2 h	Bacteria (*Acidothermus cellolyticus*)	[73]
EglII	5	50 °C	6.0	NM	Bacteria (*Bacillus amyloliquefaciens*)	[74]
EG	5	65 °C	6.0	72%; 55 °C; 42 h 50%; 65 °C; 12 min	Bacteria (*Bacillus licheniformis*)	[75]
CelA	5	60 °C	8.0	30%; 70 °C; 1 h	Bacteria (*Bacillus subtilis*)	[76]
TmCel5A	5	80 °C	6.0	50%; 80 °C; 18 h	Bacteria (*Thermotoga maritima*)	[77]
EglA	5	57 °C	4.0	NM	Fungi (*Aspergillus nidulans*)	[78]
EglB	5	52 °C	4.0	NM	Fungi (*Aspergillus nidulans*)	[78]
EBI-244	5	109 °C	5.5	50%; 100 °C; 4.5 h 50%; 105 °C; 0.57 h 108 °C; 50%; 0.17 h	Uncultured Archaea (Continental geothermal pool enrichment)	[58]
CelE1	5	50 °C	7.0	NM	Uncultured organism (Sugarcane field soil metagenome)	[64]
CelA10	5	55 °C	7.5	NM	Uncultured organism (Aquatic community and soil sample)	[66]
CelA24	5	55 °C	7.0	NM	Uncultured organism (Aquatic community and soil sample)	[66]
cMGL504	5	50 °C	5.5	NM	Uncultured organism (Vermicompost sample)	[60]
Cel5G	5	50 °C	4.8	>90%; 50 °C; 30 min	Uncultured organism (Soil metagenome)	[65]
En1	5	55 °C	5.5	87%; 45 °C; 16 h 67%; 50 °C; 6 h 42%; 55 °C; 30 min	Uncultured organism (Biogas digester metagenome)	[63]
RC1	5	55 °C	6.0–6.5	>90%; 50 °C; 30 min	Uncultured organism (Rabbit cecum metagenome)	[67]
RC3	5	50 °C	6.0–7.0	NM	Uncultured organism (Rabbit cecum metagenome)	[67]
RC5	5	50 °C	6.5–7.0	NM	Uncultured organism (Rabbit cecum metagenome)	[67]
CelL	6	50 °C	5.0	50%; 50 °C; 12 min	Bacteria (*Cellulosimicrobium funkei*)	[22]
Cel6A	6	58 °C	6.5	>80%; 56 °C; 18 h	Bacteria (*Thermobifida fusca*)	[79]
ThCel6A	6	55 °C	8.5	58%; 90 °C; 1 h	Bacteria (*Thermobifida halotolerans*)	[80]
Cel6A	6	50–55 °C	5.5	NM	Bacteria (*Xylanimicrobium pachnodae*)	[81]
HiCel6C	6	70 °C	6.5	>90%; 60 °C; 1 h	Fungi (*Humicola insolens*)	[82]
Cel6A	6	50 °C	4.8	>90%; 45 °C; 24 h 92%; 50 °C; 5 h	Fungi (*Orpinomyces* sp.)	[83]
C1	6	50 °C	6.0	100%; 60 °C; 30 min	Uncultured organism (Compost metagenome)	[61]
pre-LC-CelB	6	NM	NM	NM	Uncultured organism (Compost metagenome)	[62]
pre-LC-CelJ	6	NM	NM	NM	Uncultured organism (Compost metagenome)	[62]
EGI	7	55–60 °C	5.0	>80%; 60 °C; 10 min	Fungi (*Humicola grisea* var. *thermoidea*)	[84]
Cel7B	7	60 °C	4.0	>90%; 60 °C; 1 h	Fungi (*Penicillium decumbens*)	[85]
Cel7A	7	60 °C	5.0	100%; 60 °C; 1 h 16.1%; 70 °C; 1 h	Fungi (*Neosartorya fischeri*)	[33]
MtEG7	7	60 °C	5.0	50%; 70 °C; 9.96 h 50%; 80 °C; 6.5 h	Fungi (*Myceliophthora thermophila*)	[31]
EGL1	7	62 °C	4.8	NM	Fungi (*Trichoderma longibrachiatum*)	[51]
MaCel7A	7	65–70 °C	6.0	NM	Fungi (*Melanocarpus albomyces*)	[86]
CelC	8	50 °C	6.5	NM	Bacteria (*Salmonella typhimurium*)	[87]
Cel8Y	8	80 °C	7.0	50%; 90 °C; 4 h 50%; 100 °C; 2 h	Bacteria (*Aquifex geolicus*)	[88]
Egl-257	8	55 °C	8.5	100%; 50 °C; 15 min >50%; 60 °C; 15 min	Bacteria (*Bacillus circulans*)	[89]
CenC	9	70 °C	6.0	100%; 60 °C; 2 h 60%; 70 °C; 1 h	Bacteria (*Clostridium thermocellum*)	[90]
CelA	9 (endoglucanase) and 48 (cellobiohydrolase)	95 °C (endoglucanase) and 85 °C (cellobiohydrolase)	5.0–6.0	50%; 95 °C; 40 min (endoglucanase) 100%; 85 °C; 4 h (cellobiohydrolase)	Bacteria (*Caldicellulosiruptor bescii*)	[91]
Cel9A	9	65 °C	6.5	NM	Bacteria (*Lachnoclostridium phytofermentans*)	[92]
CelA20	9	55 °C	5.0	NM	Uncultured organism (Aquatic community and soil metagenome)	[66]
AcCel12B	12	75 °C	4.5	50%; 60 °C; 90 h 50%; 65 °C; 55 h 50%; 70 °C; 2 h	Bacteria (*Acidothermus cellulolyticus*)	[35]
CelA	12	95 °C	6.0	NM	Bacteria (*Thermotoga neapolitana*)	[8]
CelB	12	106 °C	6.0–6.6	50%; 106 °C; 130 min 50%; 110 °C; 26 min 73%; 100 °C; 4 h	Bacteria (*Thermotoga neapolitana*)	[8]
TmCel12A	12	90 °C	7.0	>40%; 85 °C; 48 h 50%; 90 °C; 3 h	Bacteria (*Thermotoga maritima*)	[93]
TmCel12B	12	85 °C	6.0	50%; 90 °C; 9 h	Bacteria (*Thermotoga maritima*)	[93]
CelA	12	>100 °C	6.0–7.0	45%; 90 °C; 8 h	Bacteria (*Rhodothermus marinus*)	[23]
EglA	12	100 °C	6.0	50%; 95 °C; 40 h	Archaea (*Pyrococcus furiosus*)	[94]
SSO1949	12	80 °C	1.8	50%; 80 °C; 8 h	Archeaea (*Sulfolobus solfataricus*)	[95]
SSO1354	12	90 °C	4.0	50%; 90 °C; 180 min	Archaea (*Sulfolobus solfataricus*)	[39]
EglS	12	65 °C	6.0	>40%; 60 °C; 30 min	Bacteria (*Streptomyces rochei*)	[96]
Cel12A	12	50 °C	5.0	NM	Fungi (*Trichoderma reseei*)	[97]
EG	12	70 °C	3.5	50%; 70 °C; 3 h 50%; 80 °C; 1 h	Fungi (*Aspergillus niger*)	[27]
Pre-LC-CelA	12	90 °C	5.0–9.0	100%; 90 °C; 30 min	Uncultured organism (Compost metagenome)	[62]
Pre-LC-CelD	12	NM	NM	NM	Uncultured organism (Compost metagenome)	[62]
Pre-LC-CelE	12	NM	NM	NM	Uncultured organism (Compost metagenome)	[62]
Cel12E	12	92 °C	5.5	>80%; 80 °C; 4.5 h	Uncharacterized archeon (deep sea vents metagenome enrichment)	[57]
GH44EG	44	55 °C	5.0	NM	Bacteria (*Clostridium acetobutylicum*)	[98]
CelA	44	60 °C	5.0–8.5	50%; 60 °C; 70 min	Bacteria (*Paenibacillus lautus*)	[99]
CelJ	44	70 °C	6.5	>90%; 80 °C; 10 min	Bacteria (*Ruminiclostridium thermocellum*)	[100]
pre-LC-CelH	44	NM	NM	NM	Uncultured organism (Compost metagenome)	[62]
Cel45A	45	60 °C	5.0	NM	Fungi (*Trichoderma reseei*)	[97]
PpCel45A	45	65 °C	4.8	70%; 65 °C; 48 h 60%; 80 °C; 4 h	Fungi (*Picchia pastoris*)	[5]
STCE1	45	60 °C	6.0	NM	Fungi (*Staphylotrichum coccosporum*)	[101]
BCC18080	45	70 °C	6.0	>70%; 70 °C; 2 h >50%; 70 °C; 4 h	Fungi (*Syncephalastrum racemosum*)	[102]
BCE1	45	55 °C	4.5	NM	Fungi (*Beltraniella portoricensis*)	[103]
MaCel45A	45	70 °C	7.0	NM	Fungi (*Melanocarpus albomyces*)	[86]
CelB	51	80 °C	4.0	60%; 80 °C; 1 h	Bacteria (*Alicyclobacillus acidocaldarius*)	[104]
CelA4	51	65 °C	2.6	>85%; 60 °C; 1 h	Bacteria (*Alicyclobacillus* sp. A4)	[47]
CelVA	51	80 °C	3.6–4.5	70%; 70 °C; 2 h	Bacteria (*Alicyclobacillus vulcanalis*)	[45]
pre-LC-CelC	51	NM	NM	NM	Uncultured organism (Compost metagenome)	[62]
TmCel74	74	90 °C	6.0	50%; 90 °C; 5 h	Bacteria (*Thermotoga maritima*)	[15]
CtCel124	124	NM	NM	NM	Bacteria (*Ruminiclostridium thermocellum*)	[105]

^1^ Temperature stability is given as a percentage of activity (residual activity) after treatment at the specified temperature and time compared to the untreated enzyme.

**Table 2:** Characterized exoglucanases (1,4-β-cellobiosidase) acting on non-reducing ends from thermophiles (EC 3.2.1.91). NM: not measured.

Enzyme	GH Family Domains	Optimum Temperature	Optimum pH	Temperature Stability ^1^	Source	Reference
CBHII	6	60 °C	4.0	30%; 100 °C; 10 min	Bacteria (*Streptomyces* sp. M23)	[106]
Cel6B	6	NM	7.0–8.0	100%; 55 °C; 16 h	Bacteria (*Thermobifida fusca*)	[107]
CBHII	6	57 °C	5.5	NM	Fungi (*Aspergillus nidulans*)	[78]
Cel6A	6	50 °C	4.0	50%; 70 °C; 30 min	Fungi (*Chaetomium thermophilum*)	[108]
CBHII (Cel6A)	6	60 °C	5.0–5.5	>90%; 50 °C; 5 h	Fungi (*Chrysosporium lucknowense*)	[109]
HiCel6A	6	60–65 °C	NM	50%; 75 °C; <25 min	Fungi (*Humicola insolens*)	[26]
Ex-4	6	50 °C	5.0	80%; 60 °C; 60 min	Fungi (*Irpex Lacteus*)	[110]
PoCel6A	6	50 °C	5.0	90%; 50 °C; 2 h 80%; 60 °C; 4 h	Fungi (*Penicillium oxalicum*)	[111]
PaCel6A	6	55 °C	5.0–9.0	100%; 35 °C; 24 h >20%; 45 °C; 24 h	Fungi (*Podospora anserina*)	[19]
CBHII	6	70 °C	5.0	NM	Fungi (*Trichoderma viride*)	[112]
G10-6	6	55 °C	9.5	NM	Uncultured organism (Eathworm casts metagenome)	[70]
Cbh9A	9	60 °C	6.5	NM	Bacteria (*Ruminiclostridium thermocellum*)	[113]
Cel9K	9	65 °C	6.0	97%; 60 °C; 200 h	Bacteria (*Ruminiclostridium thermocellum*)	[114]

^1^ Temperature stability is given as a percentage of activity (residual activity) after treatment at the specified temperature and time compared to the untreated enzyme.

**Table 3:** Characterized exoglucanases (1,4-β-cellobiosidase) acting on reducing ends from thermophiles (EC 3.2.1.176). NM: not measured.

Enzyme	GH Family Domains	Optimum Temperature	Optimum pH	Temperature Stability ^1^	Source	Reference
CelO	5	65 °C	6.6	NM	Bacteria (*Ruminiclostridium thermocellum*)	[115]
AtCel7A	7	60 °C	5.0	NM	Fungi (*Acremonium thermophilum*)	[28]
CBHI	7	60 °C	3.0	NM	Fungi (*Aspergillus aculeatus*)	[116]
CBHI	7	55 °C	NM	NM	Fungi (*Aspergillus fumigatus*)	[117]
CtCel7A	7	65 °C	4.0	NM	Fungi (*Chaetomium thermophilum*)	[28]
CBH3	7	65 °C	5.0	50%; 70 °C; 1 h 20%; 80 °C; 20 min	Fungi (*Chaetomium thermophilum*)	[118]
DpuCel7A	7	55 °C	5.0	NM	Metazoa (*Dictyostelium purpureum*)	[119]
CBHI	7	60 °C	5.0	>90%; 55 °C; 10 min	Fungi (*Humicola grisea* var. *thermoidea*)	[84]
EXO1	7	65 °C	5.0	>80%; 65 °C; 10 min	Fungi (*Humicola grisea* var. *thermoidea*)	[120]
MaCel7B	7	55 °C	NM	NM	Fungi (*Melanocarpus albomyces*)	[121]
TeCel7A	7	65 °C	4.0–5.0	50%; 70 °C; 30 min	Fungi (*Talaromyces emersonii*)	[29]
Cel7A	7	55 °C	3.7–5.2	50%; 50 °C; 2.5 h	Fungi (*Penicillium funiculosum*)	[122]
TaCel7A	7	65 °C	5.0	NM	Fungi (*Thermoascus aurantiacus*)	[28]
ThCBHI	7	50 °C	5.0	NM	Fungi (*Trichoderma harzianum*)	[123]
CBHI	7	60 °C	5.8	NM	Fungi (*Trichoderma viride*)	[112]
CelA	9 (endoglucanase) and 48 (cellobiohydrolase)	95 °C (endoglucanase) and 85 °C (cellobiohydrolase)	5.0–6.0	50%; 95 °C; 40 min (endoglucanase) 100%; 85 °C; 4 h (cellobiohydrolase)	Bacteria (*Caldicellulosiruptor bescii*)	[91]
CelY	48	70 °C	5.0–6.0	NM	Bacteria (*Clostridium stercorarium*)	[124]
CpCel48	48	55 °C	5.0–6.0	>70%; 50 °C; 30 min>20%; 55 °C; 30 min	Bacteria (*Lachnoclostridium* *phytofermentans*)	[125]
CelS	48	70 °C	5.5	NM	Bacteria (*Ruminiclostridium thermocellum*)	[126]

^1^ Temperature stability is given as a percentage of activity (residual activity) after treatment at the specified temperature and time compared to the untreated enzyme.

**Table 4:** Characterized exoglucanases (cellodextrinases) acting on reducing ends from thermophiles (3.2.1.74).

Enzyme	GH family Domains	Optimum Temperature	Optimum pH	Temperature Stability ^1^	Source	Reference
CcGH1	1	60 °C	6.5	61%; 50 °C; 30 min	Bacteria (*Clostridium Cellulolyticum*)	[127]
GghA	1	95 °C	6.5	85%; 90 °C; 9 h 88%; 95 °C; 1 h	Bacteria (*Thermotoga neapolitana*)	[128]

^1^ Temperature stability is given as a percentage of activity (residual activity) after treatment at the specified temperature and time compared to the untreated enzyme.

**Table 5:** Characterized β-glucanases from thermophiles (3.2.1.21). NM: not measured.

Enzyme	GH family Domains	Optimum Temperature	Optimum pH	Temperature Stability ^1^	Source	Reference
CelB	1	102–105 °C	5.0	50%; 100 °C; 85 h 50%; 110 °C; 13 h	Arquea (*Pyrococcus furiosus*)	[129]
Tpa-glu	1	75 °C	7.5	50%; 90 °C; 6 h	Arquea (*Thermococcus pacificus*)	[130]
BGPh	1	>100 °C	6.0	50%; 90 °C; 15 h	Arquea (*Pyrococcus horikoshii*)	[131]
LacS	1 (β-glucosidase and β-galactosidase)	90 °C	6.0	90%; 75 °C; 80 h	Arquea (*Sulfolobus solfataricus*)	[132]
O08324	1	78 °C	5.0–6.8	50%; 78 °C; 860 min	Arquea (*Thermococcus* sp.)	[133]
Bgl1	1	90 °C	6.5	67%; 90 °C; 1.5 h 78%; 50 °C; 24 h 68%; 60 °C; 24 h	Uncultured Arquea (hot spring metagenome)	[59]
GlyB	1 (multiple substrates)	85 °C	5.5	8%; 80 °C; 10 min >70%; 65 °C; 3 h	Bacteria (*Alicyclobacillus acidocaldarius*)	[134]
Bglp	1	60 °C	7.0	50%; 60 °C; 10 h	Bacteria (*Anoxybacillus flavithermus*)	[135]
BglA	1	55 °C	6.0–9.0	80%; 50 °C; 15 min 1%; 60 °C; 15 min	Bacteria (*Bacillus circulans* subsp. *Alkalophilus*)	[136]
BhbglA	1	50 °C	7.0	50%; 50 °C; 30 min	Bacteria (*Bacillus halodurans*)	[137]
BglA	1	85 °C	6.25	50%; 70 °C; 2280 min	Bacteria (*Caldicellulosiruptor saccharolyticus*)	[138]
BglA	1	50 °C	6.0	NM	Bacteria (*Clostridium cellulovorans*)	[139]
DtGH	1	90 °C	7.0	50%; 70 °C; 533 h 50%; 80 °C; 44 h 50%; 90 °C; 5 h	Bacteria (*Dictyoglomus thermophilum*)	[140]
DturβGlu	1	80 °C	5.4	70%; 70 °C; 2 h	Bacteria (*Dictyoglomus turgidum*)	[44]
FiBgl1A	1	90 °C	6.0–7.0	50%; 90 °C 25 min 50%; 100 °C; 15 min	Bacteria (*Fervidobacterium islandicum*)	[141]
BglA	1	60 °C	6.5	91%; 60 °C; 3 h 34%; 60 °C; 43 h	Bacteria (*Ruminiclostridium thermocellum*)	[142]
SdBgl1B	1	50 °C	6.0–7.5	NM	Bacteria (*Saccharophagus degradans*)	[143]
Bgl1	1	50 °C	5.1–5.7	60%; 40 °C; 4 h	Bacteria (*Sphingomonas paucimobilis*)	[144]
SGR_2426	1	69 °C	6.9	50%; 69 °C; 1.5 h	Bacteria (*Streptomyces griseus*)	[55]
Bgl3	1	50 °C	6.5	NM	Bacteria (*Streptomyces* sp. strain QM-B814)	[145]
CglT	1	75 °C	5.5	100%; 60 °C; 24 h	Bacteria (*Thermoanaerobacter brockii*)	[146]
TeBglA	1	80 °C	7.0	10%; 65 °C; 5 h	Bacteria (*Thermoanaerobacter ethanolicus*)	[147]
TmBglA	1	90 °C	6.2	>80%; 65 °C; 5 h	Bacteria (*Thermotoga maritima*)	[147]
Bgl	1	70 °C	6.4	50%; 68 °C; 1 h >80%; 60 °C; 2 h	Bacteria (*Thermoanaerobacterium thermosaccharolyticum*)	[148]
BglC	1	50 °C	7.0	NM	Bacteria (*Thermobifida fusca*)	[149]
BglB	1	60 °C	6.2	70%; 60 °C; 48 h	Bacteria (*Thermobispora bispora*)	[150]
BglA	1	80–90 °C	7.0–8.0	100%; 70 °C; 6 h	Bacteria (*Thermotoga petrophila*)	[151]
TcaBglA	1	90 °C	5.5–6.5	>40%; 80 °C; 30 min >20%; 80 °C; 30 min	Bacteria (*Thermus caldophilus*)	[152]
TnGly	1	90 °C	5.6	50%; 90 °C; 2.5 h	Bacteria (*Thermus nonproteolyticus*)	[153]
BglA	1	70 °C	5.0–6.0	50%; 70 °C; 38 h 50%; 80 °C; <0.4 h 50%; 90 °C; <0.3 h	Bacteria (*Thermus* sp. IB-21)	[154]
BglB	1	80 °C	5.0–6.0	50%; 70 °C; 38 h 50%; 80 °C; 2.7 h 50%; 90 °C; 24 min	Bacteria (*Thermus* sp. IB-21)	[154]
Bgly	1	90 °C	5.4	100%; 80 °C; 2 h 50%; 90 °C; 1.5 h 50%; 95 °C; 20 min	Bacteria (*Thermus thermophilus*)	[155]
BglA	1	55 °C	6.5	82%; 50 °C; 60 min 20%; 55 °C; 60 min	Uncultured organism (soil metagenome)	[52]
AS-Esc10	1	60 °C	8.0	100%; 50 °C; 1 h	Uncultured organism (agricultural soil metagenome)	[40]
Bgl-gs1	1	90 °C	6.0	50%; 90 °C; 5 min 50%; 85 °C; 15 min 50%; 80 °C; 45 min	Uncultured organism (termite gut metagenome)	[71]
Bgl	1	60 °C	5.0	50%; 60 °C; 540 min	Fungi (*Fusarium oxysporum*)	[156]
Bgl4	1	55 °C	6.0	80%; 50 °C; 10 min	Fungi (*Humicola grisea* var. *thermoidea* IFO9854)	[157]
Bgl1	1	55 °C	5.5–7.5	100%; 50 °C; 8 h 50%; 55 °C; 8 h	Fungi (*Orpinomyces* sp. PC-2)	[158]
Bgl1G5	1	50 °C	6.0	50%; 50 °C; 6 h	Fungi (*Phialophora* sp. G5)	[159]
TaGH2	2	95 °C	6.5	100%; 90 °C; 3 h 50%; 70 °C; 22 h	Bacteria (*Thermus antranikianii*)	[160]
TbGH2	2	90 °C	6.5	17%; 80 °C; 3 h 50%; 70 °C; 12 h	Bacteria (*Thermus brockianus*)	[160]
TbBgl	3	90 °C	3.5	50%; 95 °C; 60 min	Arquea (*Thermofilum pendens*)	[161]
BlBG3	3	50 °C	6.0	NM	Bacteria (*Bifidobacterium longum*)	[162]
Cba2	3	70 °C	4.8	NM	Bacteria (*Cellulomonas biazotea*)	[163]
CfBgl3A	3	55 °C	7.5	NM	Bacteria (*Cellulomonas fimi*)	[164]
Bgl3Z	3	65 °C	5.5	50%; 60 °C; 5 h	Bacteria (*Clostridium stercorarium*)	[165]
Dtur_0219	3	85 °C	5.0	50%; 70 °C; 1575 min 50%; 75 °C; 854 min 50%; 80 °C; 524 min 50%; 85 °C; 334 min 50%; 90 °C; 20 min	Bacteria (*Dictyoglomus* *turgidum*)	[54]
Bgl	3	50 °C	4.2–5.0	NM	Bacteria (*Elizabethkingia meningoseptica*)	[166]
TmBglB	3	80 °C	4.2	>80%; 65 °C; 5 h	Bacteria (*Thermotoga maritima*)	[147]
Tpebgl3	3	90 °C	5.0	>90%; 70 °C; 3 h >50%; 90 °C; 3 h	Bacteria (*Thermotoga petrophila*)	[167]
Cel3A	3	50–60 °C	5.0	98%; 60 °C; 6 h >50%; 60 °C; 24 h >50%; 70 °C; 24 h	Fungi (*Amesia atrobrunnea*)	[168]
Cel3B	3	50–60 °C	5.0	88%; 60 °C; 6 h >50%; 60 °C; 24 h >50%; 70 °C; 24 h	Fungi (*Amesia atrobrunnea*)	[168]
Bgl3	3	60 °C	6.0	>50%; 70 °C; 1 h	Fungi (*Aspergillus fumigatus*)	[169]
BglB	3	52 °C	5.5	NM	Fungi (*Aspergillus nidulans*)	[78]
BglC	3	52 °C	6.0	NM	Fungi (*Aspergillus nidulans*)	[78]
Bgl	3	50 °C	5.0	100%; 50 °C; 30 min 60%; 60 °C; 30 min	Fungi (*Aspergillus oryzae*)	[49]
Bgl	3	60 °C	5.0	67.7%; 60 °C; 1 h 50%; 65 °C; 55 min 29.7%; 70 °C; 10 min	Fungi (*Chaetomium thermophilum*)	[170]
Bxl5	3	75 °C	4.6	50%; 65 °C; 5 h 50%; 70 °C; 20 min 50%; 75 °C; 5 min	Fungi (*Chrysosporium lucknowense*)	[171]
MoCel3A	3	50 °C	5.0–5.5	NM	Fungi (*Magnaporthe oryzae*)	[41]
MoCel3B	3	50 °C	5.0–5.5	NM	Fungi (*Magnaporthe oryzae*)	[41]
Bgl2	3	60 °C	5.4	>50%; 40 °C; 2 h >45%; 50 °C; 2 h 25%; 55 °C; 1 h	Fungi (*Neurospora crassa*)	[172]
Bgl1	3	50 °C	3.5–5.0	100%; 45 °C; 30 min	Fungi (*Mucor circinelloides*)	[173]
Bgl2	3	55 °C	3.5–5.5	100%; 55 °C; 30 min	Fungi (*Mucor circinelloides*)	[173]
NfBGL1	3	80 °C	5.0	>80%; 70 °C; 2 h	Fungi (*Neosartorya fischeri*)	[174]
PtBglu3	3	65 °C	6.0	>85%; 60 °C; 30 min	Fungi (*Paecilomyces thermophila*)	[32]
Bgl1	3	70 °C	4.8	50%; 65 °C; 24 h	Fungi (*Penicillium brasilianum*)	[175]
pBGL1	3	65–70 °C	4.5–5.50	96.3%; 50 °C; 12 h 50%; 70 °C; 4 h	Fungi (*Penicillium decumbens*)	[176]
Bgl1	3	70 °C	5.0–6.0	60%; 70 °C; 1.5 h	Fungi (*Periconia* sp.)	[43]
RmBglu3B	3	50 °C	5.0	50%; 50 °C; 30 min	Fungi (*Rhizomucor miehei*)	[50]
Bgl1	3	50 °C	5.0	>70%; 50 °C; 30 min <10%; 60 °C; 30 min	Fungi (*Saccharomycopsis fibuligera*)	[177]
Bgl2	3	50 °C	5.0	>70%; 50 °C; 30 min <10%; 60 °C; 30 min	Fungi (*Saccharomycopsis fibuligera*)	[177]
β-glucosidase	3	75 °C	4.5	50%; 60 °C; 136 h 50%; 65 °C; 55 h 50%; 70 °C; 10 h 50%; 75 °C; 1 h	Fungi (*Talaromyces aculeatus*)	[53]
Cel3a	3	71.5 °C	4.02	50%; 65 °C; 62 min 50%; 75 °C; 18 min	Fungi (*Talaromyces emersonii*)	[178]
Bgl3A	3	75 °C	4.5	>65%; 60 °C; 1 h	Fungi (*Talaromyces leycettanus*)	[179]
Bgl1	3	70 °C	5.0	>70%; 60 °C; 1 h	Fungi (*Thermoascus auranticus*)	[180]
Bgl3a	3	70 °C	5.0	50%; 60 °C; 143 min	Fungi (*Myceliophthora thermophila*)	[181]
RG3	3	50–55 °C	5.5–6.0	NM	Uncultured organism (Rabbit cecum metagenome)	[67]
RG14	3	50–55 °C	5.5–7.0	NM	Uncultured organism (Rabbit cecum metagenome)	[67]
BGL7	3	50 °C	6.5	NM	Uncultured organism (Termite gut metagenome)	[72]
LAB25g2	3	55 °C	4.5	82%; 50 °C; 5 d	Uncultured organism (Cow rumen metagenome)	[68]
SRF2g14	3	55 °C	5.0	50%; 50 °C; 18.06 h	Uncultured organism (Cow rumen metagenome)	[68]
SRF2g18	3	50 °C	4.0	50%; 50 °C; 37.5 h	Uncultured organism (Cow rumen metagenome)	[68]
RuBGX1	3	50 °C	6.0	62%; 50 °C; 10 min	Uncultured organism (Yak rumen metagenome)	[36]
JMB19063	3	50–55 °C	6.5	NM	Uncultured organism (Compost metagenome)	[37]
GlyA1	3	55 °C	6.5	NM	Uncultured organism (Cow rumen metagenome)	[69]
Bgx1	30	50 °C	4.0–6.0	NM	Oomycota (*Phytophthora infestans*)	[182]
SSO3039	116	>70 °C	4.0	>70%; 65 °C; 48 h >50%; 85 °C; 8 h	Arquea (*Sulfolobus solfataricus*)	[183]
TxGH116	116	85 °C	6.0	NM	Bacteria (*Thermoanaerobacterium xylanolyticum*)	[184]

^1^ Temperature stability is given as a percentage of activity (residual activity) after treatment at the specified temperature and time compared to the untreated enzyme.
